# Worker Health and Safety Practices in Research Facilities Using Nonhuman Primates, North America

**DOI:** 10.3201/eid2009.140420

**Published:** 2014-09

**Authors:** Emily W. Lankau, Patricia V. Turner, Robert J. Mullan, G. Gale Galland

**Affiliations:** Centers for Disease Control and Prevention, Atlanta, Georgia, USA (E.W. Lankau, R.J. Mullan, G.G. Galland);; University of Guelph, Guelph, Ontario, Canada (P.V. Turner)

**Keywords:** Association of Primate Veterinarians, biomedical research, importation, occupational health and safety, zoonoses, nonuhman primates

**To the Editor:** Since 1975, federal quarantine regulations (*1*) have restricted nonhuman primate importation to scientific, educational, or exhibition purposes to limit risks for disease introduction (*1*,*2*). Infectious diseases resulting from importation of nonhuman primates need to be prevented to ensure that colonies of these animals are available for research and to protect persons working with them from exposure to established and emerging zoonotic diseases (*2**,**3*).

Most imported nonhuman primates are bred for research and undergo standard screening and conditioning before shipment, which substantially reduce importation-associated health risks (*4*). However, many zoonotic agents can be difficult to exclude from even meticulously controlled breeding facilities (*3**,**5*). Nonhuman primates are commonly imported from regions with a high prevalence of potentially zoonotic diseases, such as tuberculosis and meliodosis, in humans and animals. Diagnosing tuberculosis in nonhuman primates can be difficult; inadvertent colony and human exposures can occur through undiagnosed cases (*6*). Similarly, *Burkholderia pseudomallei*, the causative agent of meliodosis, can be carried asymptomatically for extended periods before illness onset, posing a persistent exposure risk for persons working with imported nonhuman primates from regions to which meliodosis is endemic (*7*). Finally, nonhuman primates are host to potentially zoonotic viruses, such as simian foamy virus, which has unknown pathogenic potential in infected persons (*8*), and Macacine herpesvirus 1, which causes severe, often fatal, neurologic disease in humans exposed to macaques with asymptomatic infection (*9*).

Quarantine and testing of imported nonhuman primates, rigorous hygiene at research facilities, and strict personal protection equipment (PPE) standards are important to protect the health of nonhuman primate colonies and persons working with the animals (*4*). Importers must register with the Centers for Disease Control and Prevention (CDC) and implement disease control measures, including a 31-day quarantine for newly arrived animals (*1*). Specific PPE is mandated for quarantine facility staff, but individual facilities determine PPE standards after the animals are released from CDC-mandated quarantine (*4*).

To better understand occupational health and safety practices at facilities housing nonhuman primates, in December 2012, the Association of Primate Veterinarians, with technical support from CDC, surveyed primate veterinarians in North America about animal handling practices and PPE standards at their institutions. The Association of Primate Veterinarians received completed surveys and removed identifying information before providing data to CDC for analysis.

CDC and the University of Guelph (Guelph, ON, Canada) determined that the survey did not qualify as human subjects research. Information collected applied to the institution, not the individual respondent. Respondents were informed that participation was voluntary and anonymous, refusal carried no repercussions, and results would be presented in aggregate.

Of 149 facilities, 7 (5%) indicated they were not currently housing nonhuman primates, and 26 (17%) provided completed surveys. Most responding facilities were university or private/contract research facilities (16 [62%] and 5 [19%] facilities, respectively). Most (18 [69%]) facilities maintained <500 nonhuman primates, primarily rhesus or cynomolgus macaques. Nineteen (73%) facilities acquired imported nonhuman primates during 2010–2012. During this period, 47,876 nonhuman primates were imported, of which 90% were cynomolgus macaques. Fewer nonhuman primates were acquired from domestic sources (1,877 animals; see also [*10*]).

In a free-text field, we asked about quarantine and testing policies for newly acquired nonhuman primates. Most facilities reported applying standard health requirements to newly acquired animals, regardless of source, and requiring additional quarantine periods before moving new animals into the facility population or assigning them to a study.

The number of staff working near nonhuman primates differed among facilities. Ten (38%) facilities reported that >30 staff members handle or work in close proximity to nonhuman primates for cleaning or observation each day (Table). All facilities required PPE for routine handling of animals, including use of surgical masks or N95 respirators; goggles, safety glasses, or full-face shields; specialized clothing (e.g., laboratory coat, scrubs, or coveralls); gloves; and either shoe covers, reusable boots, or facility-designated shoes (Table).

**Table:** Health and safety practices reported by 26 research facilities that use nonhuman primates, North America, December 2012*

Characteristic	No. (%) facilities
Average no. staff working daily with or near nonhuman primates	
1–5	8 (31)
6–10	3 (12)
11–15	1 (4)
16–20	3 (12)
21–30	1 (4)
>30	10 (38)
Required personal protection equipment*	
Respiratory protection used	
Surgical mask	21 (81)
N95 respirator	8 (31)
Powered air-purifying respirator	4 (15)
Eye protection	
Goggles/safety glasses	19 (73)
Full face shield	24 (92)
Protective clothing	
Laboratory coat/scrubs	17 (65)
Reusable coveralls	7 (27)
Disposable coveralls	15 (58)
Head covering/cap/bonnet†	8 (31)
Extra gown layer/arm covers†	5 (19)
Gloves	
Latex or nitrile gloves	26 (100)
Double gloves†	4 (15)
Leather gloves†	2 (8)
Footwear	
Shoe covers	24 (92)
Reusable boots	10 (38)
Shoes designated for use in facility only†	6 (23)
Handling of animals	
Manually capture conscious animals (“hand-catch”)‡	4 (15)
Handle conscious animals with special equipment (e.g., pole and collar, chair)	21 (81)
Conduct tuberculin skin tests on conscious animals	2 (8)
Routinely conduct necropsy on nonhuman primates that die or are euthanized§	26 (100)

*Because respondents could select >1 option, percentages will not total 100% within each personal protection equipment category. †This answer choice was not one provided in the answer options but was provided in the associated free-text field for “other.” The number provided reflects the number of respondents who volunteered this answer under “other.” ‡One respondent skipped this question. Percentages calculated with 25 facilities as the denominator. §Five respondents provided a written caveat that all animals that die spontaneously or are euthanized specifically because of health concerns routinely undergo necropsy but noted that animals euthanized at study completion often undergo study-specific tissue collection that might not include a complete necropsy.

Twenty-one (81%) facilities reported routinely handling conscious nonhuman primates by using specialized safety equipment (e.g., pole and collar or restraint chair). Four (15%) facilities reported manually capturing conscious animals (“hand-catching”); 2 (8%) facilities performed intrapalpebral tuberculin skin tests on conscious animals (Table).

All facilities reported routinely performing postmortem examinations. Five facilities specified that complete necropsies were performed only on animals found dead or euthanized because of illness or injury; for animals euthanized at study completion, targeted tissue specimens were collected to fulfill research objectives (Table).

These results suggest that responding facilities generally maintained high standards for health and safety and are aware of disease risks. However, this survey has limitations for assessing the effectiveness of risk mitigation policies. Although a variety of facilities responded, response biases cannot be excluded. Additionally, these results summarize occupational health and safety standards on record but cannot address compliance or employee attitudes toward health and safety concerns in working with nonhuman primates. Facilities maintaining nonhuman primates need to strive for strict enforcement of occupational health and safety requirements; consider requiring regular continuing education about human health risks associated with working closely with animals; and consider the degree of risk pertaining to specific activities, particularly those generating infectious aerosols.

## References

[1] Nonhuman primates. 42 CFR 71.53 (2003) [2013 cited 3 Sep]. http://www.gpo.gov/fdsys/pkg/CFR-2003-title42-vol1/xml/CFR-2003-title42-vol1-sec71-53.xml

[2] Centers for Disease Control and Prevention. Bringing a monkey into the United States [cited 2013 Sep 3]. http://www.cdc.gov/animalimportation/monkeys.html

[3] Sasseville VG, Mansfield KG. Overview of known non-human primate pathogens with potential to affect colonies used for toxicity testing. J Immunotoxicol. 2010;7:79–92. 10.3109/15476910903213521 19909217

[4] Roberts JA, Andrews K. Nonhuman primate quarantine: its evolution and practice. ILAR J. 2008;49:145–56. 10.1093/ilar.49.2.145 18323577 PMC7108595

[5] Bailey C, Mansfield K. Emerging and reemerging infectious diseases of nonhuman primates in the laboratory setting. Vet Pathol. 2010;47:462–81. 10.1177/0300985810363719 20472806

[6] Lerche NW, Yee JL, Capuano SV, Flynn JL. New approaches to tuberculosis surveillance in nonhuman primates. ILAR J. 2008;49:170–8. 10.1093/ilar.49.2.170 18323579

[7] Johnson CH, Skinner BL, Dietz SM, Blaney D, Engel RM, Lathrop GW, Natural infection of *Burkholderia pseudomallei* in an imported pigtail macaque (*Macaca nemstrina*) and management of the exposed colony. Comp Med. 2013;63:528–35 . 24326230 PMC3866985

[8] Heneine W, Switzer WM, Sandstrom P, Brown J, Vedapuri S, Schable C, Identification of a human population infected with simian foamy viruses. Nat Med. 1998;4:403–7. 10.1038/nm0498-403 9546784

[9] Huff JL, Barry PA. B-virus (*Cercopithecine herpesvirus* 1) infection in humans and macaques: potential for zoonotic disease. Emerg Infect Dis. 2003;9:246–50. 10.3201/eid0902.020272 12603998 PMC2901951

[10] Lankau EW, Turner PV, Mullan RJ, Galland GG. Nonhuman primate research use in North America. J Am Assoc Lab Anim Sci. 2014;53:278–82 . 24827570 PMC4128566

